# Allosteric Inhibitors of the NS3 Protease from the Hepatitis C Virus

**DOI:** 10.1371/journal.pone.0069773

**Published:** 2013-07-30

**Authors:** Olga Abian, Sonia Vega, Javier Sancho, Adrian Velazquez-Campoy

**Affiliations:** 1 Institute of Biocomputation and Physics of Complex Systems (BIFI), Joint Unit IQFR-CSIC-BIFI, Universidad de Zaragoza, Zaragoza, Spain; 2 IIS Aragón, Instituto Aragonés de Ciencias de la Salud, Zaragoza, Spain; 3 Centro de Investigación Biomédica en Red en el Área Temática de Enfermedades Hepáticas y Digestivas (CIBERehd), Barcelona, Spain; 4 Department of Biochemistry and Molecular and Cell Biology, Universidad de Zaragoza, Zaragoza, Spain; 5 Fundación ARAID, Government of Aragon, Zaragoza, Spain; University of Nebraska - Lincoln, United States of America

## Abstract

The nonstructural protein 3 (NS3) from the hepatitis C virus processes the non-structural region of the viral precursor polyprotein in infected hepatic cells. The NS3 protease activity has been considered a target for drug development since its identification two decades ago. Although specific inhibitors have been approved for clinical therapy very recently, resistance-associated mutations have already been reported for those drugs, compromising their long-term efficacy. Therefore, there is an urgent need for new anti-HCV agents with low susceptibility to resistance-associated mutations. Regarding NS3 protease, two strategies have been followed: competitive inhibitors blocking the active site and allosteric inhibitors blocking the binding of the accessory viral protein NS4A. In this work we exploit the intrinsic Zn^+2^-regulated plasticity of the protease to identify a new type of allosteric inhibitors. In the absence of Zn^+2^, the NS3 protease adopts a partially-folded inactive conformation. We found ligands binding to the Zn^+2^-free NS3 protease, trap the inactive protein, and block the viral life cycle. The efficacy of these compounds has been confirmed in replicon cell assays. Importantly, direct calorimetric assays reveal a low impact of known resistance-associated mutations, and enzymatic assays provide a direct evidence of their inhibitory activity. They constitute new low molecular-weight scaffolds for further optimization and provide several advantages: 1) new inhibition mechanism simultaneously blocking substrate and cofactor interactions in a non-competitive fashion, appropriate for combination therapy; 2) low impact of known resistance-associated mutations; 3) inhibition of NS4A binding, thus blocking its several effects on NS3 protease.

## Introduction

The hepatitis C virus (HCV) infection is a worldwide health problem. HCV infected people amount to more than 200 million, 80% of them becoming chronic patients, and many progressing to cirrhosis and hepatocellular carcinoma. In Europe and the United States chronic hepatitis C is the most common chronic liver disease and it is the main cause of liver transplantation. The hepatitis C infection presents serious drawbacks: 1) difficult diagnosis, asymptomatic infection and lack of preventive vaccines due to the reduced immune response against the virus; 2) severe side-effects and high cost of the current treatment leading to reduced patience adherence; 3) high natural genetic variability and appearance of drug resistance facilitated by the high replication rate together with the lack of proofreading capability in the viral RNA polymerase. Therefore, there is an urgent need for new specific, potent anti-HCV agents with reduced susceptibility to mutations in the target.

**Figure 1:**
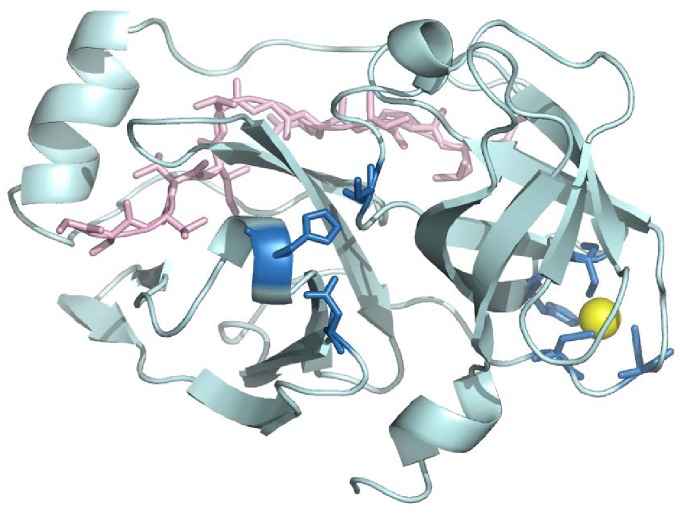
Structure of NS3 protease bound to its two cofactors, Zn^+2^ and NS4A. Crystallographic structure of the protease domain (N-terminal domain of NS3 protein) from the hepatitis C virus (PDB code: 1JXP). The two cofactors are shown: NS4A protein (pink) and zinc (yellow). The catalytic triad (H57, D81, S139) and the zinc coordination residues (C97, C99, C145, H149) are shown in blue sticks. Dissociation of the NS4A protein in the N-terminal domain leads to partial unfolding of that domain and slight distortion of the catalytic triad spatial configuration. Dissociation of the zinc atom in the C-terminal domain causes global unfolding in both domains.

NS3 protease is a 20 KDa serine protease structurally homologous to other extracellular serine proteases, such as trypsin and chymotrypsin, located at the N-terminal domain of the NS3 protein. Homologous extracellular proteases present disulfide bridges stabilizing the molecular structure. However, as expected for an intracellular protease working under reducing conditions, NS3 does not contain disulfide bridges, but a Zn^+2^ ion tetra-coordinated by three cysteine residues and a histidine residue located in its C-terminal domain [1]–[4] (Figure 1). The Zn^+2^ ion is required for the hydrolytic activity, since its removal leads to inactivation. However, it is located very far (>20 Å) from the catalytic triad (H57/D81/S139 in NS3 numbering) to be directly involved in catalysis. Consequently, the Zn^+2^ ion is considered to have a structural, stabilizing role equivalent to that of the disulfide bonds present in other serine proteases. NS3 protease function additionally requires the binding of the viral nonstructural protein 4A (NS4A) [5]–[8], which provides further structural stabilization through restructuring the N-terminal domain of NS3 protease, enhancement of the proteolytic activity by changing the configuration of the catalytic triad of NS3 protease, and appropriate cellular membrane localization through a highly hydrophobic terminal NS4A portion. Both NS4A and Zn^+2^ enhance the catalytic efficiency of the protease. While NS3 protease presents some basal level of proteolytic activity in the absence of NS4A, it has no activity in the absence of Zn^+2^.

Since its identification, the NS3 protease has been considered a pharmacological target for drug discovery. Although a broad variety of competitive inhibitors has been developed, very few of them have entered clinical trials. Very recently, two protease inhibitors have been approved by the FDA for therapeutic treatment [9], [10]. However, resistance mutations causing an efficacy reduction for these two drugs have already been identified [11]–[13]. Some efforts have been directed at developing allosteric inhibitors that directly block the NS4A binding site [14]–[16].

A detailed biophysical characterization of the NS3 protease suggested a considerable global conformational change upon Zn^+2^ binding involving mainly the tertiary structure of the protein [17], [18]. Disruption of the NS3-Zn^+2^ interaction leads to significant unfolding into a conformation resembling a molten globule and maintaining some residual structure. The conformational change will move apart the catalytic residues, since H57 and D81 are located in the N-terminal domain, and S139 in the C-terminal domain, leading to enzyme inactivation. Therefore, in the absence of Zn^+2^, NS3 protease is mostly unstructured and can be considered an intrinsically (partially) disordered protein that folds upon binding to its Zn^+2^ cofactor. The conformational stability of the Zn^+2^-free NS3 protease is very low (Gibbs energy of stabilization at 20°C of 0.4 kcal/mol), and fully unfolded protein (35%) coexists with partly unstructured-folded protein (65%) at that temperature. Thus, Zn^+2^ binding provides most of the stabilization energy in the Zn^+2^-bound NS3 protease. Therefore, regarding the Zn^+2^ interaction, three structurally, energetically and functionally distinguishable conformational states can be considered: the Zn^+2^-bound folded native protein, the Zn^+2^-free unstructured partly folded protein, and the completely unfolded protein. NS3 protease being an intracellular enzyme, its structural molecular integrity is regulated by the free Zn^+2^ pool inside the cell. There is some controversy about the concentration of free Zn^+2^ within the cell, but normal levels are in the low nanomolar range [19]. As soon as the internal concentration of free Zn^+2^ slightly increases, regulatory processes are initiated in order to remove any Zn^+2^ excess [20]–[22]. According to this accepted hypothesis, cells do not operate with a significant pool of free Zn^+2^ and cellular Zn^+2^ homeostasis and occupancy are under kinetic control. In addition, it has been shown that, similarly to most Zn^+2^ enzymes, the interaction of NS3 protease with Zn^+2^ exhibits very slow kinetics, with a small kinetic association constant [23].

The complex allosterically modulated conformational landscape of the NS3 protease should not be regarded as peculiar. In a broad sense, allostery consists in the modulation of protein conformational equilibrium and function by ligand binding. Since any ligand binding preferentially to a given conformational state will stabilize it and increase its population, the population of the different conformational states of a protein will depend on their interaction with certain ligands. In this way, ligands concentration may control protein function through modulation of the protein conformational landscape. The pH dependency of binding interactions and enzymatic activities in proteins is perhaps the most pervasive type of allosteric control: proton binding to certain functional groups modulates the population of different protein conformational and functional states.

In this work, we exploit the complex conformational landscape of the NS3 protease for developing new allosteric inhibitors. The existence of an inactive partially-folded highly-populated conformation for NS3 protease in the absence of Zn^+2^, significantly dissimilar to the active native state, led us to the establish the following hypothesis: because the population of Zn^+2^-free inactive partially-folded state is physiologically relevant, specific ligand binding will stabilize it and trap the enzyme into that conformation, preventing the activating effect of the interaction with its Zn^+2^ and NS4A cofactors, thus, effectively inhibiting the catalytic activity in an allosteric fashion. The tightly regulated low intracellular Zn^+2^ pool may contribute to the efficacy of this new allosteric inhibition mechanism.

Inhibiting the enzyme by shifting the conformational equilibrium from a properly folded Zn^+2^-bound enzyme to a partially folded Zn^+2^-free inactive enzyme as a result of ligand binding must be energetically costly and kinetically very unfavourable (large activation energy). But, interfering with and disrupting the timing of the processes leading to an active enzyme may be plausible. Thus, in addition to equilibrium interaction parameters, kinetic considerations should be taken into account for the different processes involved: protein synthesis and folding, Zn^+2^ interaction, intracellular Zn^+2^ concentration. Because all these processes are tightly coupled, the trapping of the protein into an inactive conformational state due to ligand binding together with a low intracellular Zn^+2^ concentration may represent a considerable barrier towards producing an active enzyme.

To identify compounds exhibiting this behaviour, an experimental ligand screening was performed following NS3 protease stabilization under conditions where the inactive partially-folded conformation is predominantly populated (in the presence of a saturating concentration of ethylene diamine tetraacetic acid, EDTA, a Zn^+2^-chelating agent). A set of compounds were selected based on the stabilizing effect induced on NS3 protease against thermal denaturation. Their potency as HCV-replication inhibitors and their cytotoxicity were evaluated in cell assays. These inhibitors show a new mechanism of action in which substrate and cofactor interactions are blocked in a non-competitive fashion, show a low binding affinity reduction against resistance-associated NS3 protease mutant variants, and could be used in combination therapy with the current approved clinical inhibitors.

## Materials and Methods

### Chemical Library

The compounds were supplied dissolved in dimethyl sulfoxide (DMSO) 100% at a concentration of 4 mM (Prestwick Chemical, USA). According to the manufacturer, the compounds are FDA-approved drugs selected for their high chemical and pharmacological diversity. In addition, information on their bioavailability, as well as toxicity and safety, in humans is available.

### NS3 Protease

The N-terminal domain from full-length NS3 protein corresponding to NS3 protease was expressed and purified as described elsewhere [17], [18]. The isolated protease domain exhibits similar properties (enzymatic activity, inhibition constants, and allosteric activation mechanism) to those of the full-length protein [24].

### Cells and Replicon System

The highly permissive cell clone Huh 7-Lunet, as well as Huh 7 cells containing subgenomic HCV replicons I389luc-ubi-neo/NS3-3′/5.1 (Huh 5-2), I377NS3-3′/wt (Huh 9-13) or I389/hygro-ubi-NS3-3/5.1 (a kind gift from Dr. V. Lohmann and Dr. R. Bartenschlager) have been described recently [25]-[28]. Cells were grown in Dulbecco’s modified Eagle’s medium (DMEM; Gibco, Belgium) supplemented with 10% heat-inactivated fetal bovine serum (PAN-Biotech GmbH, Germany), 1X non-essential amino acids (Gibco), 100 IU/mL penicillin (Gibco), 100 µg/mL streptomycin (Gibco), and 250 µg/mL geneticin (G418; Gibco).

### Experimental Screening

A non-native inactive partially-folded conformation of the NS3 protease is populated in the absence of Zn^+2^. Although this state is highly unstructured, it maintains a significant amount of residual structure and may be stabilized by ligands. Ligands binding to and stabilizing this inactive conformation will act as allosteric inhibitors trapping the NS3 protease into an inactive conformation.

Ligands for NS3 protease have been identified by an experimental screening procedure based on a thermal-shift assay similar to that employed previously for identifying small-molecule compounds acting as *Helicobacter pylori* flavodoxin inhibitors and human phenylalanine hydroxylase chaperones [29], [30]. Ligands targeting the inactive partially-folded NS3 protease state have been identified as those compounds inducing a stabilizing effect in NS3 protease against thermal denaturation in the absence of Zn^+2^. The protein unfolding transition was monitored by following ANS (8-anilino 1-naphthalene sulfonic acid; Sigma-Aldrich) emission fluorescence, which greatly increases when it binds to hydrophobic regions in the protein exposed to the solvent upon thermal unfolding.

Ligand-induced NS3 stabilization was assessed by monitoring the thermal denaturation of recombinant pure NS3 protease in a FluoDia T70 High Temperature Fluorescence Microplate Reader (Photon Technology International, UK). Protein-ligand solutions (100 µL) were dispensed into 96-well microplates (ThermoFast 96 skirted plates, Thermo Scientific) and overlaid with 20 µL of mineral oil to prevent evaporation. Protein solutions contained 2 µM NS3 protease in 100 mM sodium acetate, 2 mM EDTA, pH 5, and 100 µM ANS. Ligands dissolved in DMSO were added at 100 µM (with a final 2.5% residual concentration of DMSO) to microplates containing the protein solutions and incubated at 25°C for 30 minutes before loading into the microplate reader. Control experiments with NS3 protease samples with/without DMSO and/or Zn^+2^ were routinely performed in each microplate. Thermal denaturation was monitored by following the increase in ANS fluorescence intensity associated with protein unfolding (λ_exc_ = 395 and λ_em_ = 500 nm, where λ_exc_ is the excitation wavelength and λ_em_ is the emission wavelength^27^). Unfolding curves were registered from 25°C to 75°C in 1°C steps. The system was allowed to equilibrate at each temperature for 1 minute before each fluorescence acquisition. In practice, this represents an operational heating rate of 0.25°C/min approximately.

Although in the absence of Zn^+2^ NS3 retains some structure, it shows very low stability against thermal denaturation [18]. Approximately 40% of the molecules are completely unfolded at 25°C. Therefore, the native baseline in the pre-unfolding region is absent in the thermal denaturation assays. The mid-transition temperature can be operationally defined as the temperature for maximal slope in the unfolding curve or, alternatively, the temperature at which half of the maximal change in the signal is achieved. The absence of the native pre-unfolding baseline makes somewhat difficult the evaluation of the mid-transition temperature following the second criterion. Therefore, hits were identified as those compounds shifting the temperature for maximal slope towards higher temperatures, compared to the internal controls in each microplate.

### Antiviral Assay with Huh 5-2 Cells

Antiviral assays for assessing the efficacy of the selected compounds were performed as described in literature [23]–[28]. Briefly, Huh 5-2 cells were seeded at a density of 5⋅10^3^ cells per well in a tissue culture-treated white 96-well view plate (Techno Plastic Products AG, Switzerland) in complete DMEM supplemented with 250 µg/mL G418. After incubation for 24 hours at 37°C medium was removed and 2-fold serial dilutions in complete DMEM (without G418) of the test compounds were added in a total volume of 100 µL. After 3 days of incubation at 37°C cell culture medium was removed and luciferase activity was determined using the Bright-Glo™ Luciferase Assay System (Promega Corporation, The Netherlands). The luciferase signal was measured using a Synergy HT Multimode Reader (BioTek Instruments Inc, USA). The 50% effective concentration (EC50) was defined as the concentration of compound that reduced the luciferase signal by 50%.

### Cytostatic Assay

Cytostatic assays for assessing the cell viability of the selected compounds were performed as described in literature [25]–[28]. Briefly, Huh 5-2 were seeded at a density of 5⋅10^3^ cells per well of a 96-well plate in complete DMEM with the appropriate concentrations of G418. Serial dilutions of the test compounds in complete DMEM (without G418) were added 24 hours after seeding. Cells were allowed to proliferate for 3 days at 37°C, after which the cell number was determined by CellTiter 96 AQ_ueous_ One Solution Cell Proliferation Assay (Promega Corporation). The 50% cytostatic concentration (CC50) was defined as the concentration that inhibited the proliferation of exponentially growing cells by 50%.

### Isothermal Titration Calorimetry (ITC) Assay

Ligand binding to NS3 protease was determined with a high-sensitivity isothermal titration VP-ITC microcalorimeter (MicroCal, USA). Protein samples and reference solutions were properly degassed and carefully loaded into the cells to avoid bubble formation during stirring. Experiments were performed with freshly prepared buffer-exchanged protein solutions, at 25°C in 100 mM sodium acetate, 2 mM EDTA, pH 5. NS3 protease 20 µM solution in the calorimetric cell was titrated with compound 300 µM solution. Control experiments were performed under the same experimental conditions, except for the presence of zinc (no addition of EDTA). The heat evolved after each ligand injection was obtained from the integral of the calorimetric signal. The heat due to the binding reaction was obtained as the difference between the reaction heat and the corresponding heat of dilution, the latter estimated as a constant heat throughout the experiment, and included as an adjustable parameter in the analysis. The association constant (K_a_) and the enthalpy change (ΔH) were obtained through non-linear regression of experimental data to a model for a protein with a single ligand binding site. Data were analyzed using software developed in our laboratory implemented in Origin 7 (OriginLab, USA).

NS3 protease wild-type, pseudo-wild-type (containing the inactivating mutation S139A) and several variants containing drug-resistance associated mutations (R155K, R155Q, A156T, and D168A) were employed for determining the impact of these mutations on the interaction of the selected compounds with the protein (change in dissociation constant).

### In vitro Enzymatic Inhibition Assay

The inhibition effect of the selected compounds on NS3 protease was assessed employing the FRET substrate Ac-Asp-Glu-Asp(EDANS)-Glu-Glu-Abu-L-lactoyl-Ser-Lys(DABCYL)-NH_2_ (Bachem AG, Switzerland) [31]. A solution of purified NS3 protease at 1 µM final concentration (buffer sodium acetate pH 5) was treated sequentially adding: 1) EDTA at 100 µM final concentration; 2) compound at 0 or 25 µM final concentration (compound stock solutions are 10 mM in 100% DMSO; therefore, the same volume of DMSO was added to the control samples without compounds); 3) Zn^+2^ at either 200, 100, or 50 µM final concentration. Each addition of EDTA, compound and Zn^+2^ was followed by 30-minute incubation at room temperature. Finally, substrate was added at 4 µM final concentration for initiating the hydrolysis reaction. Fluorescence intensity was measured in triplicates using a Synergy HT Multimode Reader (BioTek Instruments Inc, USA) using 380 nm and 500 nm for excitation and emission wavelengths, respectively. NS3 protease activity was determined as the initial slope of the curve. The quotient between the activity in the presence (25 µM) and the absence of a given compound provides the percentage of inhibition.

## Results

### Identification of NS3 Protease Ligands by Experimental Screening

The presence of ANS and DMSO might induce an additional minor destabilization, but the observed unfolding traces are in agreement with those obtained in the absence of DMSO and ANS by monitoring the intrinsic tryptophan fluorescence in NS3 protease [18]. Besides, the minor destabilizing effect induced by ANS and DMSO will be the same for all samples, including the controls.

**Figure 2:**
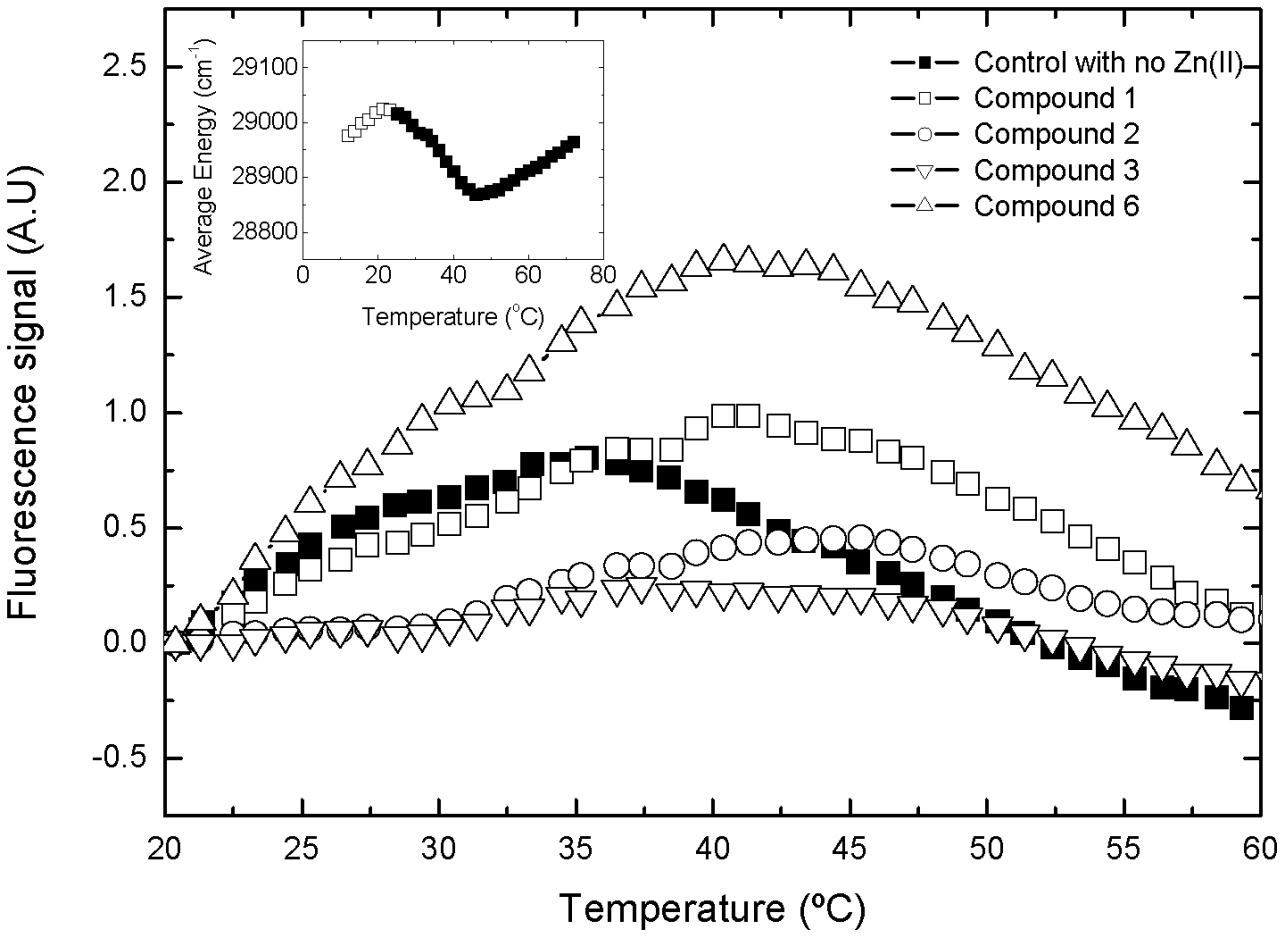
Experimental screening for ligands binding to the Zn^+2^-free NS3 protease. Thermal denaturation curves of Zn^+2^-free NS3 protease followed by ANS fluorescence in the presence of different compounds (100 mM sodium acetate, pH 5, 2 mM EDTA). Inset: Thermal denaturation of Zn^+2^-free NS3 protease followed by tryptophan intrinsic fluorescence (average energy of spectra). The unfolding transition restricted to the temperature range accessible in the microplate fluorescence reader for the library screening is indicated in closed squares for comparison.

**Figure 3:**
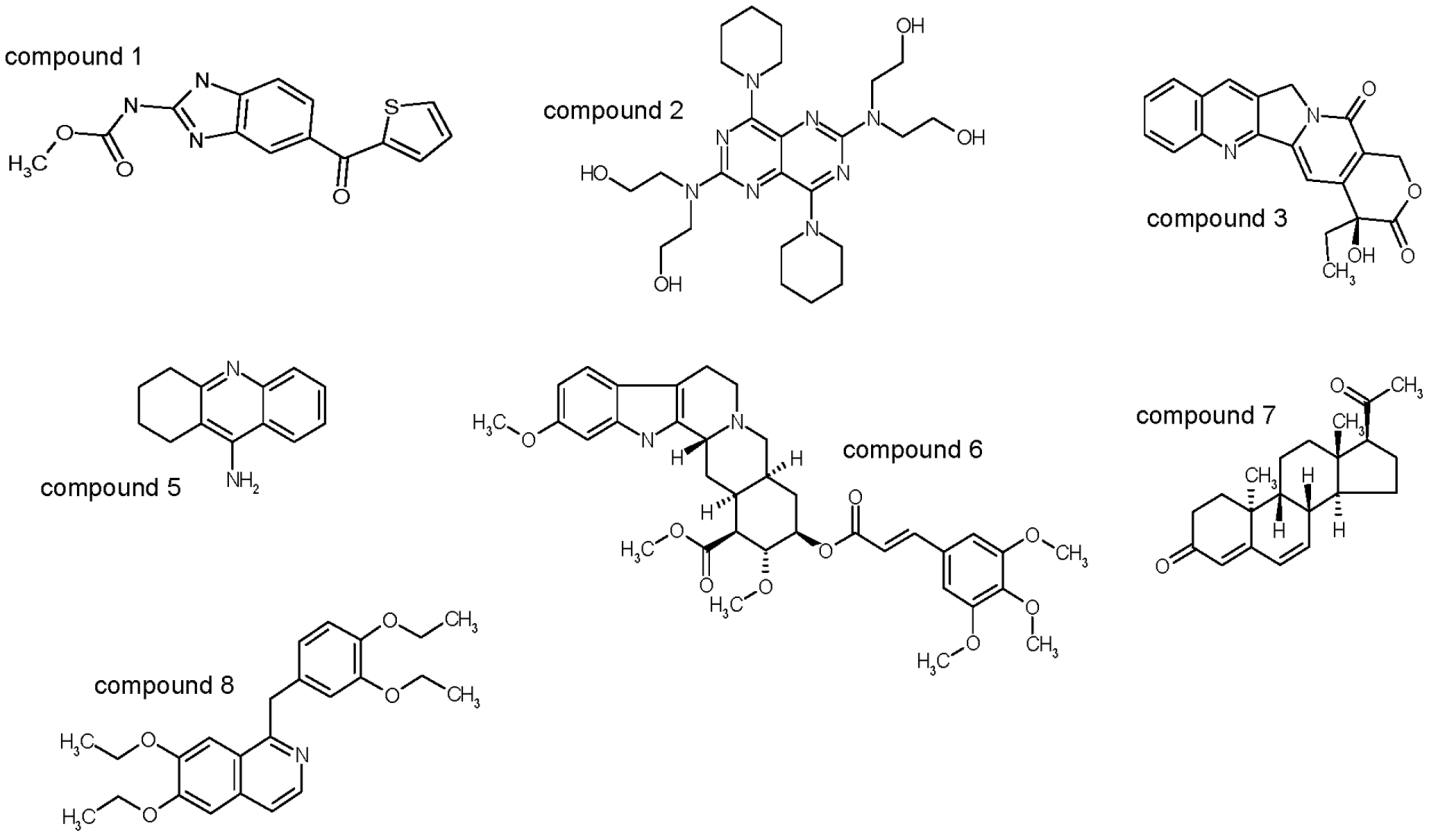
Compounds selected from experimental screening and exhibiting antiviral activity in cell assays. Chemical structures of the selected compounds by experimental screening were further tested in cell-based, calorimetric and enzymatic assays.

Although the Zn^+2^-free NS3 protease retains some structure, it shows very low stability against thermal denaturation. Approximately 40% of the molecules are unfolded at 25°C. Therefore, the native baseline in the pre-unfolding region is absent in the thermal denaturation assays (Figure 2). Compounds binding to the NS3 protease are identified as those compounds stabilizing the protein against thermal unfolding, i.e. those compounds increasing the mid-transition temperature. The mid-transition temperature has been defined as the temperature for maximal slope in the unfolding curve. Therefore, hits were identified as those compounds shifting the temperature for maximal slope to higher temperatures, compared to the internal controls in each microplate (Figures 2 and 3).

### Antiviral Activity of Selected Compounds in HCV Subgenomic Replicon Cells

Compounds selected in the experimental screening are ligands targeting the inactive partially-folded Zn^+2^-free NS3 protease conformation. Therefore, those compounds will act as allosteric inhibitors stabilizing and trapping the NS3 protease into an inactive conformation.

**Figure 4:**
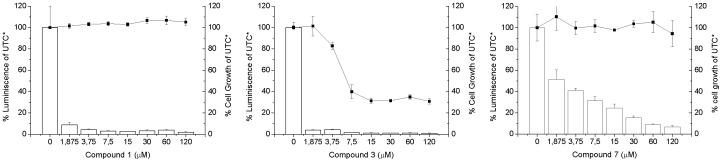
Inhibition of HCV replicon in cell assays. Evaluation of potency and cytotoxicity of the selected compounds in cell assays. HCV replicon replication rate (white bars) and cell survival (closed squares) were assessed in cell culture at increasing compound concentration to determine EC50 and CC50. For compounds 1 and 3 assays with lower concentrations were performed in order to reliably determine the EC50. *UTC: untreated controls.

In order to test if this hypothesis is correct and the compounds effectively inhibit viral replication, the potency of these compounds was assessed by *in vitro* HCV replication assays with genotype 1b Con1 HCV subgenomic replicons (Huh 5-2). From the 17 compounds identified from the thermal up-shift screening, 7 of them inhibited HCV replicon replication (measured as luciferase signal) in a dose-dependent manner with EC50 lower than 100 µM (Table 1, Figure 4). The estimated EC50 values for these compounds ranged from nanomolar to micromolar. The anti-HCV activity was not the result of a cytostatic effect, since the CC50 values for these compounds were significantly higher than the EC50 values.

**Table 1:** Evaluation of potency and cytotoxicity of the selected compounds in cell assays.

	EC50 (µM)^a^	CC50 (µM)^b^	CC50/EC50
Compound 1	0.03	>120	>4000
Compound 2	10	80	8
Compound 3	0.03	8	240
Compound 5	60	>120	>2
Compound 6	20	>120	>6
Compound 7	2	>120	>60
Compound 8	4	60	15

a EC50, effective concentration 50%;

b CC50, cytotoxic concentration 50%.

Relative error in the parameters is 15%.

### Binding of Selected Compounds to Wild Type and Drug-resistance NS3 Protease Variants by ITC

**Figure 5:**
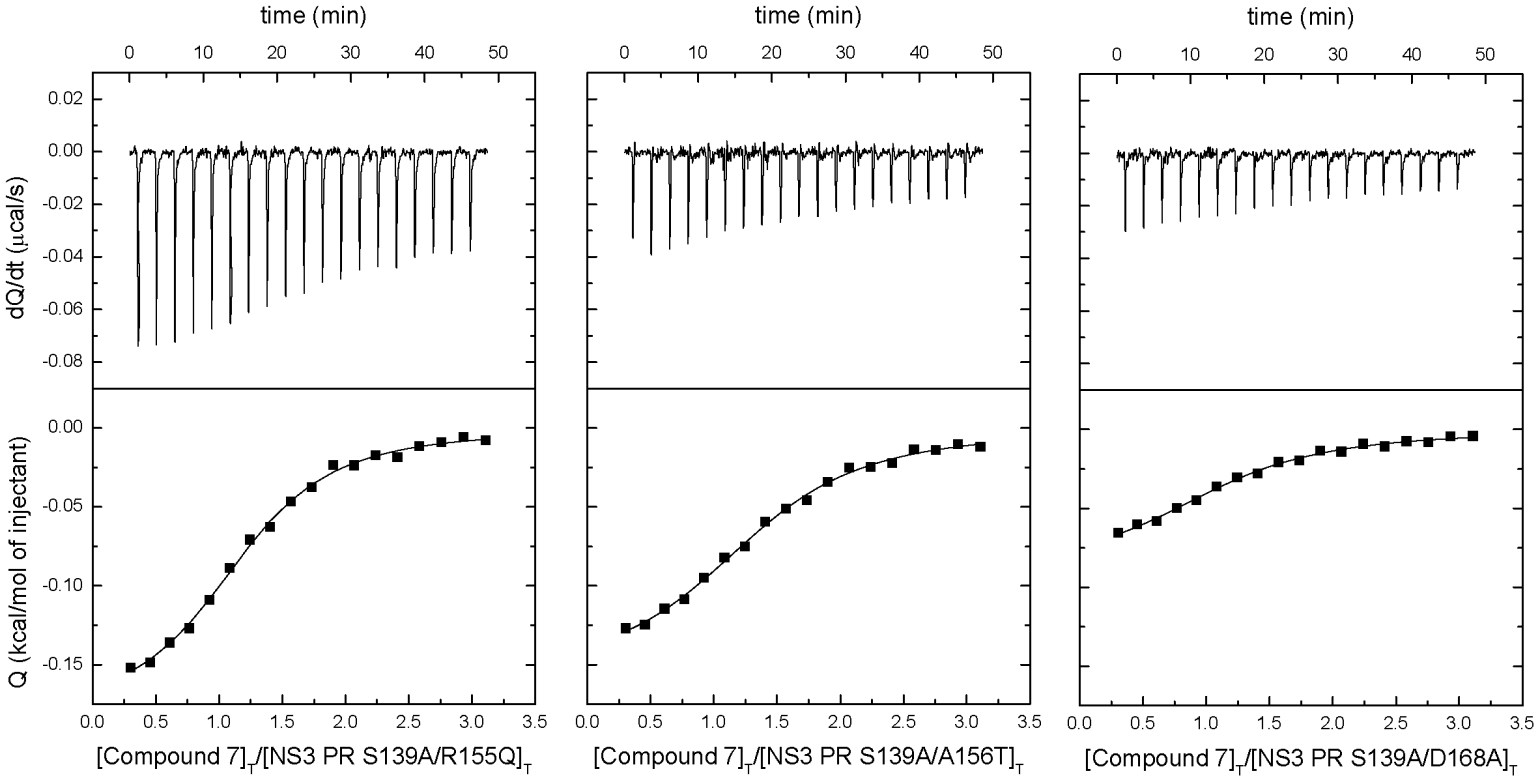
Interaction with Zn^+2^-free NS3 protease. Calorimetric assays for determining the binding affinity of selected compounds to the NS3 protease. Upper plots: thermograms representing the thermal power required for maintaining the calorimetric cell at constant temperature; lower plots: binding isotherm representing the heat associated with each injection as a function of the advance of the binding process ([ligand]_Total_/[protein]_Total_). The continuous line corresponds to the non-linear regression fitting analysis considering a single binding site model.

The dissociation constants for the interaction between NS3 protease wild-type, pseudo-wild-type (S139A) and drug-resistance associated variants with the selected compounds are summarized in Table 2. The results indicate that these compounds interact specifically with the Zn^+2^-free NS3 protease conformation. All NS3 variants tested bind these compounds with similar affinity, with a dissociation constant (K_d_) in the micromolar range (Figure 5) and K_d_ ratios no larger than 5 (compared to the pseudo-wild-type NS3). Therefore, the impact of the currently known resistance-associated mutations is small. Control experiments in the presence of Zn^+2^ (no EDTA added) indicated the compounds do not bind to the Zn^+2^-bound protein.

**Table 2:** Dissociation constants for selected compounds binding to drug-resistance-associated NS3 protease variants (25°C, 100 mM sodium acetate, 2 mM EDTA, pH 5).

	K_d_ (µM)						
	WT	S139A^a^	S139A/R155K	S139A/R155Q	S139A/A156T	S139A/D168A	
Compound 1	1.9	6.5	5.3			2.6	
Compound 2	29	8.4	23	21		11	
Compound 3	2.5						
Compound 5	1.7	5.5	5.9	32	27	8.5	
Compound 6	40	24	61	23	45	36	
Compound 7	16	5.7	16	4.2	5.8	6.5	
Compound 8	31	34	9.2	42	22	16	

a The inactive S139A mutant represents the pseudo-wild-type, behaving similarly to wild-type (WT) NS3 protease in terms of structural stability, substrate binding affinity and NS4A activation.

Relative error in the parameters is 15%.

### Inhibition of NS3 Protease

The direct inhibition of NS3 protease was tested with the selected compounds. The protocol consisted of: 1) removing Zn^+2^ from the enzyme by adding EDTA (to obtain Zn^+2^-free NS3 protease); 2) adding a given inhibitor compound (to the Zn^+2^-free NS3 protease); 3) adding Zn^+2^ to the enzyme; 4) and initiating the catalytic reaction by adding the substrate. According to the dissociation constant for the EDTA-Zn^+2^ at pH 5 (K_d_ of 0.8 nM [14]), and the total concentrations of Zn^+2^ (50, 100 and 200 µM) and EDTA (100 µM) employed in the inhibition assay, the concentration of free Zn^+2^ for each case is 0.8 nM, 0.9 µM and 0.1 mM, covering 5 orders of magnitude.

**Figure 6:**
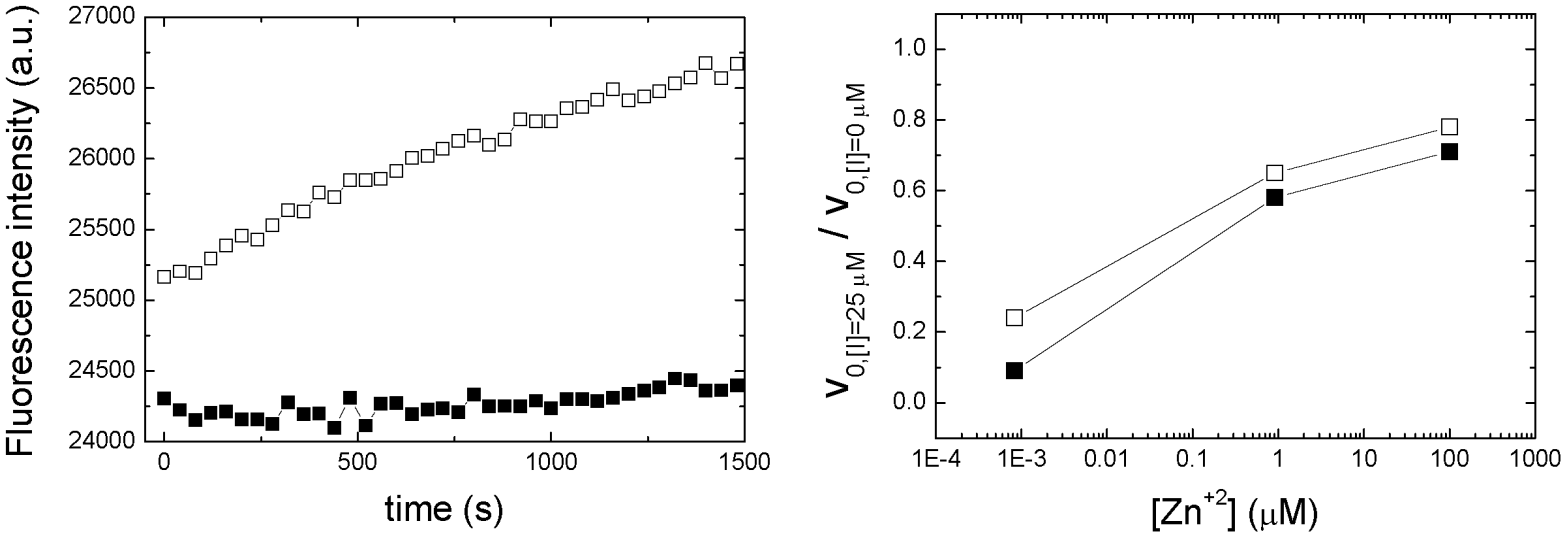
In vitro enzymatic inhibition of NS3 protease. (Left panel) Fluorescence intensity measured as a function of time (wavelengths of 380 nm and 500 nm for excitation and emission, respectively) for the substrate catalysis by NS3 protease in the absence (open squares) or presence of compound 1 at 25 µM (closed squares), in sodium acetate pH 5, 0.8 nM free Zn^+2^ concentration. (Right panel) NS3 protease activity was determined as the initial slope of the curves. The percentage of activity is calculated as the quotient between the activity of NS3 protease in the presence (25 µM) and the absence of a given compound (compound 1, closed squares; compound 5, open squares).

At a given concentration of Zn^+2^, the presence of the selected compounds at 25 µM concentration reduces the catalytic activity of the NS3 protease (Figure 6). The percentage of inhibition (ratio between the initial rates with and without compound) depends on Zn^+2^ concentration. At normal intracellular concentration there is significant inhibition, whereas at high concentration inhibition capability is significantly reduced (Figure 6). Similar results were obtained with all the selected compounds.

## Discussion

Drug development against hepatitis C virus targeting the NS3 protease with competitive inhibitors has proven difficult mainly due to a very shallow substrate binding site. Binding to a solvent-exposed binding site does not benefit enough from the solvent entropy gain originated by water molecules release upon binding, and rely in the optimization of different intermolecular interactions (hydrogen bonds, van der Waals and electrostatic interactions) for achieving sufficient affinity. Thus, despite the vast amount of knowledge gathered on serine proteases during the last several decades, drugs targeting the NS3 protease consisting of competitive inhibitors have only been recently approved. As expected in the case of a virus with high replication rate and lack of proofreading ability, resistance mutations have been already reported for these drugs [11]–[13]. Targeting the NS3-NS4A interaction has also experienced limited success. Therefore, there is an urgent need for new approaches and strategies for drug development against HCV.

The biophysical characterization of a protein target provides invaluable information on: 1) structural and functional features of the protein target; 2) potential strategies and methodologies for ligand binding screening; 3) criteria for optimizing ligand binding affinity and specificity. The NS3 protease is a small serine protease with a complex conformational landscape modulated by its two natural cofactors, NS4A and Zn^+2^. In the absence of its two natural cofactors, it is unable to fold into its active conformation. Zn^+2^ binding is responsible for 60% of structural organization and at least 80% of the stabilization energy of the native folded conformation [15], [18]. Therefore, NS3 protease folding is strongly coupled to the interaction with a Zn^+2^ ion, and in the absence of Zn^+2^ the NS3 protease adopts a partially folded inactive conformation with some residual structure. This conformation is relevant *in vivo* considering that NS3 is synthesized intracellularly within the viral polyprotein, and must be processed and folded appropriately into the active conformation in an environment experiencing Zn^+2^ stress, where intracellular Zn^+2^ concentration is very low, in the nanomolar range or lower, and it is tightly, kinetically regulated [19]–[22]. In fact, the association kinetics for Zn^+2^ binding to the NS3 protease is considerably slow. In particular, an apparent association rate constant, k_obs_, of around 0.014 s^−1^ can be estimated at pH 7.5 [23]. If a pseudo-first order association can be assumed, would yield a k_on_ <0.0014 µM^−1^s^−1^. Under physiological conditions, the intracellular zinc concentration is in the low nanomolar range (or even lower) and, therefore, the rate of association would be lower than 0.000014 s^−1^. Moreover, through refolding experiments of NS3 protease by measuring the catalytic activity in the presence of substrate and increasing concentrations of zinc, an apparent dissociation constant of about 1 µM for the NS3-zinc interaction can be estimated [1]. In addition, by performing ITC experiments, a dissociation constant of 0.5 µM for the NS3-zinc interaction was estimated at pH 5 [17].

Consequently, the Zn^+2^-free conformation of the NS3 protease is structurally and energetically distinguishable from the folded active conformation and represents a new drug target. Any ligand specifically binding to that alternative conformation will stabilize and trap such inactive conformation, thus allosterically inhibiting the enzyme.

An experimental screening based on ligand-induced stabilization against thermal denaturation (thermal up-shift) provided several candidate compounds. This methodology allowed selecting small molecules able to bind to the Zn^+2^-free conformation of the NS3 protease. Despite some common structural features (e.g. presence of aromatic rings), there is low structural similarity between these compounds. The targeted conformation of the protein (Zn^+2^-free NS3 protease) is highly flexible due to structure loss and can be considered as an ensemble of energetically indistinguishable conformational microstates. Different ligands may bind to different structural motifs in the partly unfolded protein.

Replicon cell assays were performed in order to validate the initial experimental screening. Some of the identified compounds were able to inhibit the replication of the replicon system (Figure 3, Table 1). In these *in vitro* assays, additional processes besides direct enzyme-inhibitor interaction are taking place: internalization and membrane pump efflux of compounds, synthesis and processing of the viral polyprotein (in particular, full-length NS3), viral replication cycle, and hepatic cell metabolism, among others.

Calorimetric titrations were conducted to assess the direct enzyme-inhibitor interaction. The compounds were able to bind to the Zn^+2^-free NS3 protease with moderate-to-high binding affinities (dissociation constants in the micromolar range or lower) (Table 2). There is no need for very high affinities for these compounds, because they will not compete with the substrate or the allosteric activator NS4A for their respective binding site. There is no direct correlation between dissociation constant (K_d_) and EC50 values. However, the dissociation constant is determined from biophysical assays using purified protein and reflects the direct interaction of each compound with the Zn^+2^-free conformation of the NS3 protease, while the EC50 is determined from cell-based assays and reflects the inhibition activity on the replicon system involving several coupled processes as mentioned before (protein synthesis, protein folding, ligand binding, ligand availability, etc.).

The selected compounds will hinder the conformational transition between two different conformations, one largely unfolded and another native-like folded, in an allosteric fashion, and, therefore, these compounds will block simultaneously the binding of substrate and NS4A. In addition, the impact of reported resistance mutations associated to current clinical NS3 protease inhibitors is quite small (not larger than 5-fold). Again, this is due to the fact these selected compounds are directed towards a different target (Zn^+2^-free NS3 protease conformation) compared to the current clinical competitive inhibitors (Zn^+2^-bound NS3 protease). There might be other mutations affecting the binding of these compounds, but they must be different from the already reported resistance mutations.

Continuous enzymatic assays using a FRET substrate provided direct evidence for the inhibition activity of the selected compounds on NS3 protease. The inhibition assays were performed at different Zn^+2^ concentrations, from values under typical intracellular Zn^+2^ concentrations to very high values. Zn^+2^ promotes folding of the enzyme towards the folded active conformation, whereas the selected compounds trap the enzyme into the inactive partially folded conformation. As expected, as a result of the competing action exhibited by the compounds and Zn^+2^ (though they are not competitive inhibitors), the percentage of inhibition decreases with Zn^+2^ concentration.

**Figure 7:**
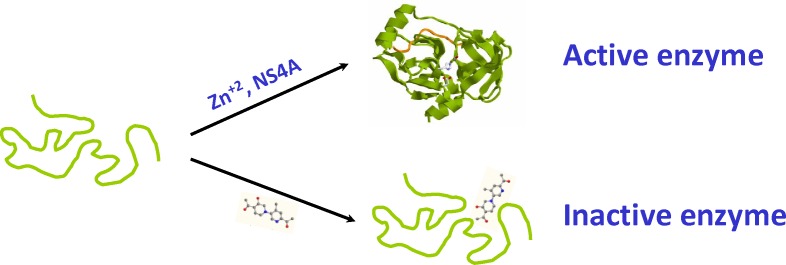
Action mechanism of the allosteric inhibitors. The proper folding of the NS3 protease towards the active conformation, promoted *in vivo* by its two cofactors (Zn^+2^ and NS4A), is prevented by the compounds through stabilization of the inactive partially folded Zn^+2^-free conformation.

Traditionally, allosteric effectors (activators or inhibitors) have been considered to exert their action on well-defined structures, modulating the populations of the different conformational states according to their intrinsic binding affinities and their effective concentrations. The selected compounds described in this work bind specifically to a conformational state of the target that is largely unstructured with some residual structure, preventing the conformational transition towards the active folded conformation (Figure 7). In fact, there is no requirement for residual structure for a given target to constitute a drug target, since the ligand binding may induce the structural rearrangement, at least locally, and block the target by preventing further interactions. In addition, the targeted conformational state may be transiently populated, as long as it is accessible enough for efficient ligand binding. Therefore, this work highlights the importance of alternative partially-folded conformational states, even transiently populated, in pharmacological targets for drug design and development strategy. The approach shown here for the NS3 protease might be of outstanding relevance bearing in mind the large number of metalloproteins (in which the structural integrity of the protein is strongly coupled to the interaction with a metal ion characterized by low bioavailability) and of intrinsically (partially) disordered proteins in genomes.

The advantages of these new allosteric inhibitors can be summarized as follows: 1) new inhibition mechanism in which substrate and cofactor interactions are simultaneously blocked in a non-competitive fashion; 2) new low molecular weight scaffolds for further optimization (regarding affinity, specificity, toxicity,…); 3) reduced impact of known resistance-associated mutations, showing a small affinity reduction for drug-resistant NS3 protease variants; 4) inhibition of NS4A binding, blocking its several effects on NS3 protease (e.g. activity enhancement, intracellular membrane localization within the replicative complex); and 5) possibility of combination therapy (highly-active antiviral therapy) together with the current approved clinical inhibitors.

### Conclusions

New allosteric inhibitors of the NS3 protease have been identified by exploiting the fairly complex conformational landscape of NS3 protease. The intrinsic plasticity of the enzyme coupled to the tight regulation of Zn^+2^ intracellular concentration at very low levels give rise to an inactive partially-folded conformational state in the absence of Zn^+2^. This alternative conformational state can be considered a new drug target, and compounds binding to it will stabilize and trap the protein into that inactive partially-folded state, therefore, blocking the viral life cycle (Figure 7). The efficacy and cytotoxicity of these compounds have been evaluated in cell assays, and they show promising characteristics: low molecular weight, moderate-to-high potency (from micromolar to nanomolar EC50) and moderate-to-low cytotoxicity. Besides, these inhibitors show a new mechanism of action, and could be combined with current competitive clinical inhibitors to develop a highly-active anti-viral combination therapy. On the other hand, these compounds are FDA-approved drugs for different therapeutic indications (compound 1: antineoplastic agent; compound 2: inhibitor of thrombus formation; compound 3: antineoplastic agent; compound 5: cholinergic and parasympathomimetic agent, s; compound 6: antihypertensive agent; compound 7: progestin agonist; and compound 8: parasympatholytic agent), and valuable information regarding pre-clinical tests (e.g. pharmacokinetic data) is available.

Future work will address resistance susceptibility and cross-resistance patterns for the identified compounds, compared to known NS3 inhibitors, by generating replicon systems containing resistance-associated mutations.
